# Perioperative multiple low-dose Dexamethasones improves postoperative clinical outcomes after Total knee arthroplasty

**DOI:** 10.1186/s12891-018-2359-1

**Published:** 2018-12-01

**Authors:** Yuangang Wu, Xiaoxi Lu, Yimei Ma, Yi Zeng, Xianchao Bao, Huazhang Xiong, Bin Shen

**Affiliations:** 1 Department of Orthopaedic Surgery, West China Hospital, West China Medical School, Sichuan University, Chengdu, 610041 Sichuan Province China; 2 Department of Pediatrics, West China University Second Hospital, Sichuan University, Chengdu, 610041 Sichuan Province China; 3 Key Laboratory of Birth Defects and Related Diseases of Women and Children (Sichuan University), Ministry of Education, Chengdu, China

**Keywords:** Total knee arthroplasty, Dexamethasones, Clinical outcomes, Randomized controlled study

## Abstract

**Background:**

The purpose of this study was to investigate the efficacy and safety of multiple low-dose dexamethasones in primary total knee arthroplasty (TKA).

**Methods:**

One hundred fifty patients were equally randomized into 3 groups: Group A (*n* = 50) received 2 doses of normal saline only; Group B (*n* = 50) received with 1 dose of intravenous dexamethasone and 1 dose of normal saline; Group C (*n* = 50) received with 2 doses of intravenous dexamethasone. The clinical outcomes and complications were assessed.

**Results:**

The CRP and IL-6 were significantly lower in Group C and B than Group A at 24, 48, and 72 h postoperatively (*P* < 0.001 for all). The intensity of postoperative nausea and vomiting (PONV) in Group C was lower than Group A at 24 (*P* < 0.001, *P* = 0.002), 48 (*P* = 0.005, *P* = 0.041) and 72 h (*P* = 0.017, *P* = 0.031) postoperatively and Group B at 24 h (*P* = 0.027, *P* = 0.019) postoperatively. Pain were significantly less in Group C than Group A at 24 (*P* < 0.001), 48 h (*P* = 0.037) postoperatively and Group B 24 h (*P* = 0.030) postoperatively. Patients in Group C had better range of motion (ROM) and satisfaction than Group A (*P* < 0.001, *P* = 0.002) and B (*P* = 0.001, *P* = 0.043). No differences were found in complications.

**Conclusions:**

The administration of 10 mg dexamethasone 1 h before the surgery, and repeated at 6 h postoperatively can significantly reduce the level of postoperative CRP and IL-6 and the incidence of PONV, relieve pain, achieve an additional analgesic effect, and improve the early ROM compared with the other two groups in TKA.

**Level of Evidence:**

Therapeutic Level I.

**Trial registration:**

The Chinese Clinical Trial Registry (ChiCTR1800017036). Registered on July 9, 2018.

## Background

Total knee arthroplasty (TKA) has become a successful surgical method for the treatment of severe knee diseases [1–3]. TKA, however, involves extensive osteotomy, soft tissue release and surgical trauma, which often lead to severe postoperative inflammatory reactions [4–6]. Consequently, patients can experience severe pain in the early postoperative period [7, 8], accompanied by postoperative nausea and vomiting (PONV) [9–11], and a prolonged length of stay (LOS) [12]. Pain and PONV may hinder the early recovery of TKA, resulting in patients dissatisfaction [13–16]. Therefore, it is better to improve the fast-track recovery of patients in reducing postoperative inflammation, relieving pain and preventing PONV.

Glucocorticoids, which have powerful anti-inflammatory and antiemetic effects, are widely used in perioperative management, such as abdominal [17, 18], gynaecologic [19], and TKA surgery [20–22], for reducing the postoperative inflammatory response, alleviating postoperative pain and preventing PONV. However, the most suitable protocol for administration and dosage remains controversial. As previously reported, glucocorticoids were given at a single and low dose, with fewer samples in most previous studies [23–25]. In addition, concerns regarding the adverse effects of glucocorticoid therapy, including infection and gastrointestinal bleeding, have potentially limited its widespread use in TKA [26]. Last, although the administration of the drug provides better pain and vomiting relief than traditional methods, many patients still suffer from it at the initial stage after surgery [19, 22, 27].

The current study was therefore designed to compare the effectiveness and safety of multiple low doses of dexamethasone in patients following primary TKA by evaluating: (1) whether multiple low doses of dexamethasone reduce postoperative inflammatory markers and the incidence of PONV; (2) whether multiple low doses of dexamethasone relieve pain and provide an additional analgesic effect, improving the range of motion (ROM) and patient satisfaction; and (3) whether multiple low doses of dexamethasone are safe in primary TKA.

## Methods

### Study design and patients

This randomized controlled trial was approved by the institutional ethics committee and written informed consent was obtained from each patient. The study was registered in the Chinese Clinical Trial Registry on 09/07/2018 (ChiCTR1800017036). All consecutive patients with a diagnosis of end-stage osteoarthritis following primary unilateral TKA were enrolled for inclusion in the study. The exclusion criteria were as follows: rheumatoid arthritis, revision surgery, allergy to the dexamethasone and tranexamic acid, administration of the glucocorticoid 3 months before surgery, alcohol dependence, a history with thrombosis, the severe liver and kidney deficiency and body mass index (BMI) > 35 kg/m^2^. Patients were randomly assigned to three groups containing 50 patients with a list generated by a computer, and randomization was blind and performed with the use of sealed envelopes at a ratio of 1:1:1 to be opened just prior to surgery.

Patients in the Group A was given intravenously 2 mL of normal saline solution 1 h before the surgery and repeated 6 h after surgery. Patients in the Group B was given intravenously 10 mg dexamethasone (2 ml, Tianjin Kingyork group Co., Ltd., China) 1 h before the surgery and repeated intravenously 2 mL of normal saline solution 6 h after surgery. Patients in the Group C was given intravenously 10 mg dexamethasone solution 1 h before the surgery and repeated 6 h after surgery. To support the double-blind study, patients in group A were treated with 2 doses of a normal saline solution and patients in group B were treated also normal saline solution 6 h after surgery. All drugs were administered by a nurse who was not involved in the study. The patients, investigators, and statisticians were all blind during the study.

### Surgical technique

All patients were performed general anesthesia, which was administered by the same anesthetists. All TKAs were performed by 1 senior orthopedic surgeon through a midline skin incision, medial parapatellar approach. All patients have used the PFC Sigma PS (DePuy Orthopedics Inc., Warsaw, IN, USA) prosthesis. All of the wound closed at about 45°of knee bends. The arthrotomy closure was performed using an interrupted figure-of-eight #1 EthibondTM (Ethicon, Somerville, NJ, USA), a subdermal closure using interrupted buried simple 2–0 Monocryl TM (Ethicon) and staples for skin closure. All patients were given intraoperatively 20 mg/kg tranexamic acid (Chongqing Lummy Pharmaceutical Co., Ltd. China) 10 min before the skin incision, and repeated 3 h after surgery. No patients used the tourniquet, drainage tube, femoral nerve block and/or intravenous patient-controlled analgesia.

### Postoperative care

All patients received cefuroxime 1.5 g 2 h before the operation to prevent infection. After the operation, the patient was transferred to the anesthesia recovery unit for 2 h and then sent to the inpatient ward. A cold pack was applied to the surgical site 2 days. Daily gait rehabilitation program and weight training were conducted by a physiotherapist on the first day after surgery.

All patients received the same management for pain and PONV. Multimodal oral analgesic drugs (200 mg q12 h celecoxib, 75 mg q8 h pregabalin) were administered for pre-emptive analgesia 1 day before the surgery. After the surgery, the pain levels of all patients were measured using a visual analogue scale (VAS, 0 - no pain, 10 - worst imaginable pain). The routine analgesia regimen remains the same as before surgery when the VAS level of the patient was lower than 4. Oral oxycodone (10 mg q8 h) was used when the VAS level of the patient was between 4 and 6, and intramuscular injection of pethidine hydrochloride (100 mg) was used when a patient reported pain greater than 6. The severity of nausea of all patients was measured using VAS (VAS, 0-on nausea, 10- worst imaginable nausea). Metoclopramide (10 mg) was injected intravenously as the first-line antiemetic rescue treatment when PONV occurred two or more times or the VAS level was greater than 4. An intramuscular injection of ondansetron (5 mg) can be used as the second-line antiemetic rescue option when severe nausea persists after two doses of metoclopramide for a 30-min interval.

All patients were given subcutaneously low molecular-weight heparin (LMWH, 0.2 mL, 2000 IU) at 6 h postoperatively, and repeated with a full dose at 24-h intervals (0.4 mL, 4000 IU) until discharge. After discharge, 10 mg rivaroxaban (Xarelto, Bayer, Germany) was administered orally for 15 days to prevent thrombosis [28]. Intermittent pneumatic compression device was routinely applied on the calves of patients until walking. Doppler ultrasound examination was used assessing deep vein thrombosis (DVT) at the time of discharge and at 1, 3-month follow-up assessments, or at any time clinically suspected DVT. Pulmonary embolism (PE) was diagnosed on the basis of clinical symptoms and chest computed tomography (CT) scans.

### Outcome measurements

The outcomes were evaluated including the inflammation reaction of CRP and IL-6, pain level (VAS score) and the number of patients requiring analgesic rescue drugs (Oxycodone and Pethidine hydrochloride), the severity of nausea (VAS score), the incidence of PONV and the number of patients requiring antiemetic rescue (Metoclopramide and Ondansetron). CRP, IL-6, pain level, the severity of nausea, and PONV were routinely tested preoperatively and at 24, 48, and 72 h postoperatively. Nausea was defined as a subjective feeling of unpleasant related to awareness of the urge to vomit. Vomiting is the forcible discharge of stomach contents from the mouth. ROM and a six-point satisfaction questionnaire [5] was assessed at the time of discharge. The LOS and wound-related complications were recorded carefully.

### Statistical analysis

The sample size of the current study was calculated, as previously described by Lunn [24], using VAS score as the primary outcome; it was determined that, for 90% power and a significance level of 0.05, requiring 24 patients in each group. With the consideration of exclusion, we decided to include 50 patients in each group. One-way ANOVA and Tukey’s post-hoc were used to compare the quantitative data, such as CRP, IL-6, pain level, the severity of nausea, and ROM. The Pearson chi-square test or Fisher exact test was used for comparing qualitative data, such as the incidence of PONV, patient satisfaction, and complications. All analyses were performed using SPSS version 22.0, significance was set at *P* < 0.05.

## Results

### Baseline characteristics

**Fig. 1:**
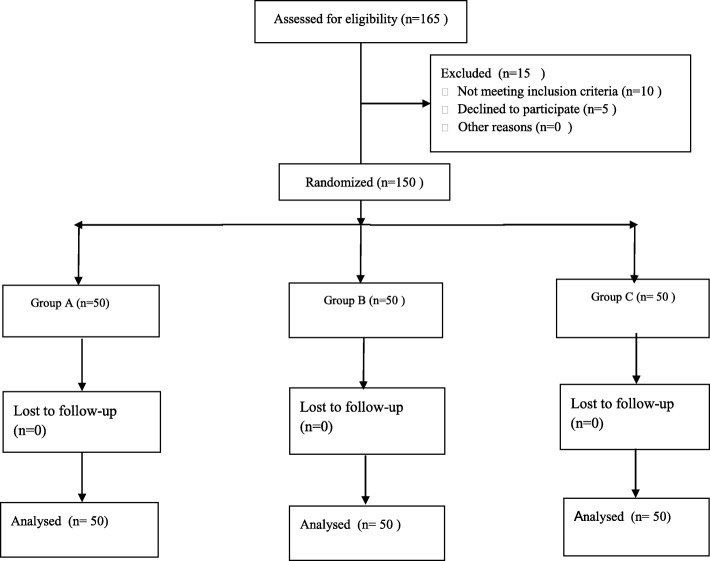
A flow diagram shows the patients assessed and included among 3 groups

During the recruitment period from January 2017 to October 2017, 165patients with osteoarthritis requiring unilateral primary TKA were scheduled. Among these patients, 15 of these patients were excluded for the following reasons: 6 patients with glucocorticoid 3 months before surgery, 3 had alcohol dependence, 5 declined to participate, 1 had an infection. Thus, 150 patients were eventually included in the analysis, 50 patients were randomized to each group (Fig.1). No patients were lost during the follow-up. Table 1 summarizes the baseline characteristics of the 3 groups. There were no statistically significant differences in age, gender, BMI, American Society of Anesthesiologists, preoperative Hb, Hct, CRP, IL-6, ROM and VAS among the 3 groups of patients.

**Table 1:** Preoperative demographics

Variables	Group A (*n* = 50)	Group B (*n* = 50)	Group C (*n* = 50)	*P* Value
Age (y)^*^	67.40 ± 3.34	66.90 ± 4.62	66.38 ± 3.38	0.414
Gender^‡^ (M/F)	32/18	33/17	30/20	0.818
Weight (kg)^*^	65.52 ± 4.99	65.22 ± 4.81	66.38 ± 3.84	0.423
Hight (cm)^*^	159.38 ± 6.76	158.06 ± 5.81	157.92 ± 6.86	0.465
BMI (kg/m^2^)^*^	25.85 ± 2.08	26.14 ± 1.98	26.70 ± 2.08	0.109
ASA^*^	1.98 ± 0.65	1.94 ± 0.65	2.02 ± 0.62	0.824
Preop.Hb (g/L)^*^	13.42 ± 0.55	13.45 ± 0.53	13.39 ± 0.56	0.858
Preop.Hct (L/L)^*^	39.84 ± 1.26	39.87 ± 1.17	39.74 ± 1.25	0.867
Preop.CRP (mg/L)^*^	3.23 ± 0.96	3.29 ± 0.76	3.25 ± 0.93	0.950
Preop.IL-6 (pg/mL)^*^	4.05 ± 1.10	4.15 ± 1.15	4.11 ± 0.47	0.859
Preop. ROM^*^	94.54 ± 3.40	93.90 ± 3.42	93.89 ± 4.15	0.594
Preop. VAS^*^	5.16 ± 0.68	5.32 ± 0.82	5.22 ± 0.68	0.542
Duration of surgery (min)^*^	66.84 ± 3.30	67.92 ± 3.30	68.02 ± 2.83	0.120

Abbreviations**:** *y* years, *M* male, *F* female, *R* right, *L* left, *BMI* body mass index, *ASA* American Society of Anesthesiologists, *Preop* preoperative, *Hb* hemoglobin, *Hct* hematocrit, *CRP* C-reactive protein, *IL-6* interleukin 6, *ROM* range of motion, *VAS* visual analogue scale

*P* value indicates a significant difference among the groups

*was analyzed by the one-way ANOVA;

‡ was analyzed by the Pearson chi-square test or the Fisher exact test

### Inflammation marks

**Fig. 2:**
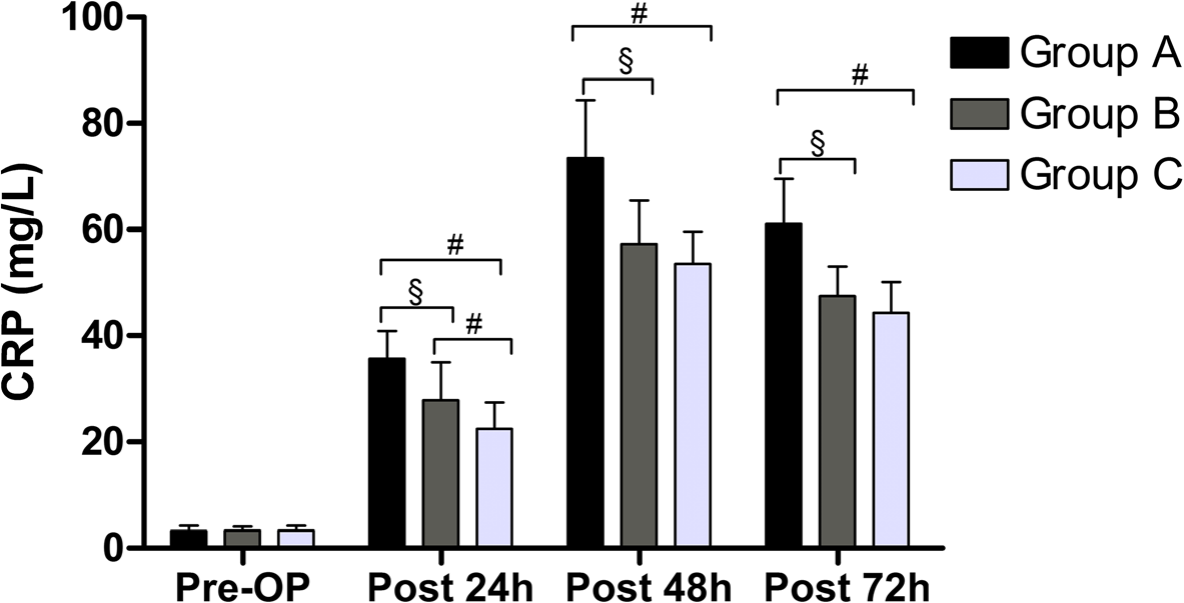
The level of CRP in all groups. Pre-OP = preoperative, post = postoperative. A significant difference among the 3 groups as calculated with one-way ANOVA. ‡ Significantly different from the Group B. # Significantly different from the Group C

The mean CRP and IL-6 increased postoperatively in all patients after the surgery. The mean level of CRP peaked 48 h postoperatively among the 3 groups. It was significantly lower in Group C and Group B than Group A at 24 (*P* < 0.001, *P* < 0.001), 48 (*P* < 0.001, *P* < 0.001), and 72 h (*P* < 0.001, *P* < 0.001) postoperatively. The mean level of CRP in Group C was lower than Group B at 24 h (*P* < 0.001) postoperatively, however, it was no statistical significance at 48 (*P* = 0.081) and 72 h (*P* = 0.057) postoperatively (Fig.2).

**Fig. 3:**
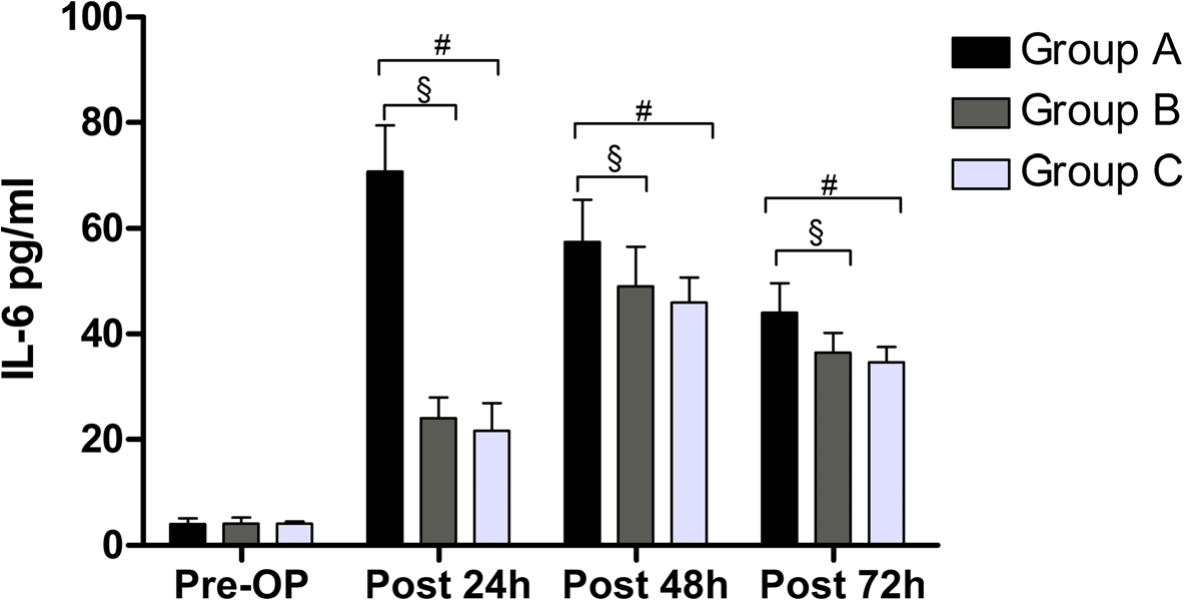
The level of IL-6 in all groups. Pre-OP = preoperative, post = postoperative. A significant difference among the 3 groups as calculated with one-way ANOVA. ‡ Significantly different from the Group B. # Significantly different from the Group C

The mean level of IL-6 peaked in Group C and B at 48 h postoperatively compared with Group A at 24 h postoperatively. It was significantly lower in Group C and Group B than Group A at 24 (*P* < 0.001, *P* < 0.001), 48 (*P* < 0.001, *P* < 0.001), and 72 h (*P* < 0.001, *P* < 0.001) postoperatively. Although, the mean level of IL-6 in Group B was slightly greater than Group C at 24 (*P* = 0.133), 48 (*P* = 0.073), and 72 h (*P* = 0.075) postoperatively, it was no statistical significance (Fig.3).

### Pain level and rescue analgesic

**Fig. 4:**
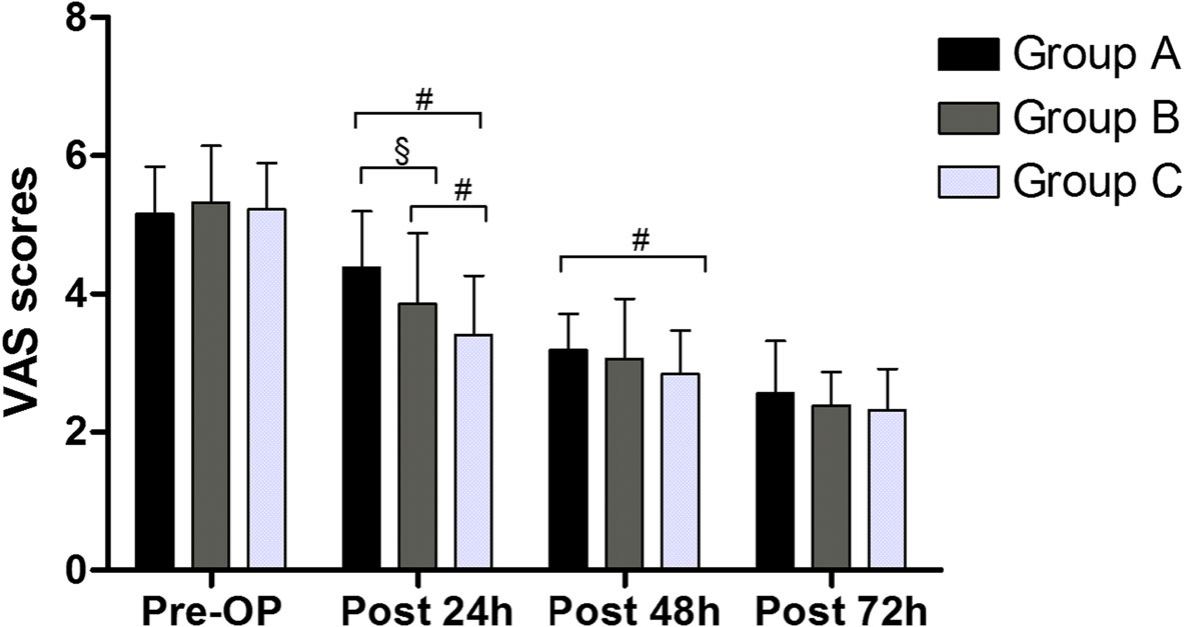
The level of pain in all groups. Pre-OP = preoperative, post = postoperative. A significant difference among the 3 groups as calculated with one-way ANOVA. ‡ Significantly different from the Group B. # Significantly different from the Group C

The postoperative mean VAS pain score was significantly lower in Group C than in Group A at 24 h (*P* < 0.001), 48 h (*P* = 0.037) postoperatively and compared with B 24 h (*P* = 0.030) postoperatively. The differences were also statistically significant between Groups B and A at 24 h (*P* = 0.012) postoperatively (Fig. 4).

Similar to the pain level, the number of patients requiring oxycodone and pethidine hydrochloride was lower in Group C compared with Group A at 24 h (*P* < 0.001, *P* < 0.001) and 48 h (*P* = 0.003, *P* < 0.001) postoperatively, and it was also lower in Group B than Group A at 24 h postoperatively (*P* = 0.027, *P* = 0.034). The level of pethidine hydrochloride was less in Group C than B at 24 h postoperatively (*P* = 0.003) (Table 2).

**Table 2:** The the number of patients of requiring rescue analgesic and antiemetic

Variables	Group A (*n* = 50)	Group B (*n* = 50)	Group C (*n* = 50)	*P* Value
Oxycodone-post 24 h (n)^‡^	28§#	17 #	8	0.001
Oxycodone-post 48 h (n)^‡^	19 #	11	6	0.009
Oxycodone-post 72 h (n)^‡^	8	5	3	0.265
Pethidine hydrochloride-post 24 h (n)^‡^	22§#	12	6	0.001
Pethidine hydrochloride-post 48 h (n)^‡^	14 #	7	2	0.004
Pethidine hydrochloride-post 72 h (n)^‡^	3	1	0	0.166
Metoclopramide-post 24 h (n)^‡^	21§#	10	7	0.003
Metoclopramide-post 48 h (n)^‡^	12 #	7	3	0.039
Metoclopramide-post 72 h (n)^‡^	6 #	2	0	0.025
Ondansetron-post 24 h (n)^‡^	7	3	1	0.064
Ondansetron-post 48 h (n)^‡^	2	0	0	0.132
Ondansetron-post 72 h (n)^‡^	0	0	0	1

Abbreviations**:** *post* postoperative, *h* hours, *n* number

‡ was analyzed by the Pearson chi-square test or the Fisher exact test

P value indicates a significant difference among the groups

§Significantly different from the Group B. # Significantly different from the Group C

### Intensity of nausea, PONV, and rescue antiemetic

The intensity of nausea in Group C was lower than Group A at 24 (*P* < 0.001), 48 (*P* = 0.005) and 72 h (*P* = 0.017) postoperatively and compared with Group B at 24 h (*P* = 0.027) postoperatively, and it was also lower in Group B than Group A at 24 h postoperatively (*P* = 0.041) (Table 3).

**Table 3:** The clinical outcomes and complications among the 3 groups

Variables	Group A (*n* = 50)	Group B (*n* = 50)	Group C (*n* = 50)	*P* Value
Intensity of Nausea-post 24h^*^	2.96 ± 1.24§#	2.38 ± 1.07#	1.76 ± 1.24	0.001
Intensity of Nausea-post 48h^*^	1.66 ± 0.82#	1.38 ± 0.90	1.12 ± 0.82	0.008
Intensity of Nausea-post 72h^*^	1.18 ± 0.77#	0.94 ± 0.73	0.74 ± 0.85	0.023
PONV-post 24 h^‡^	22§#	11	8	0.004
PONV- post 48 h^‡^	14 #	8	5	0.058
PONV- post 72 h^‡^	10 #	4	2	0.026
Satisfaction level (n)^‡^				0.007
Very satisfied	18 #	23 #	35	
Somewhat satisfied	14	17	11	
Neither satisfied nor dissatisfied	11	6	4	
Somewhat dissatisfied	7	4	0	
Very dissatisfied	0	0	0	
ROM^*^	99.66 ± 2.60 #	100.70 ± 2.37 #	102.48 ± 1.99	0.001
LOS (days)^*^	5.02 ± 0.62	4.94 ± 0.84	4.82 ± 0.72	0.393
DVT (n)^‡^	0	0	0	1
PE (n)^‡^	0	0	0	1
Intramuscular thrombosis (n)^‡^	3	3	5	0.675
Superficial infection (n)^‡^	0	1	2	0.360
Gastrointestinal hemorrhage (n)^‡^	0	0	0	1

Abbreviations: *Post* postoperative, *h* hours, *n* number, *PONV* postoperative nausea and vomiting, *ROM* range of motion, *LOS* length of stay, *DVT* deep vein thrombosis, *PE* Pulmonary embolism

*was analyzed by the one-way ANOVA;

‡ was analyzed by the Pearson chi-square test or the Fisher exact test

P value indicates a significant difference among the groups

§Significantly different from the Group B. # Significantly different from the Group C

Similar to the intensity of nausea, the incidence of PONV was lower in Group C than Group A at 24 (*P* = 0.002), 48 (*P* = 0.041) and 72 h (*P* = 0.031) postoperatively, and there was also a significant difference between Groups B and A at 24 h (*P* = 0.019) postoperatively (Table 3).

The number of patients requiring metoclopramide was lower in Group C compared with Group A at 24 (*P* < 0.001), 48 (*P* = 0.025) and 72 h (*P* = 0.035) postoperatively, and it was also lower in Group B than Group A at 24 h (*P* = 0.017) postoperatively (Table 2).

No three-group differences in the number of patients requiring ondansetron were found at 24 (*P* = 0.064), 48 (*P* = 0.132) and 72 h (*P* = 1) postoperatively (Table 2).

### ROM, LOS, patient satisfaction, and complications

At the time of discharge, the ROM in Group C was better than in Group A (*P* < 0.001) and B (*P* = 0.001), but it was no differenced in the other subgroup in another subgroup. The average LOS in Group A, B, C were 5.02 ± 0.62, 4.94 ± 0.84, and 4.82 ± 0.72 days respectively, no differences were found (*P* = 0.393). Patients in Group C had significantly higher satisfaction ratings than Groups A (*P* = 0.002) and B (*P* = 0.043) (Table III), no benefits in Group B relative to Group A was found (*P* = 0.298) (Table 3).

No DVT and PE was found in any of the patients, however, 9 patients (group A, 3 cases, group B, 3 cases, group C, 5 cases) developed intramuscular venous thrombosis. 2 patients from Group C and 1 patients from Group B had superficial infection during the 3-month follow-up period, which was controlled by dressing change and oral antibiotics. No gastrointestinal hemorrhage occurred (Table 3).

## Discussion

The most important finding of this study is that patients in Group C who were intravenously administered 10 mg dexamethasone 1 h preoperatively and repeated 10 mg at 6 postoperatively can significantly reduce the postoperative level of CRP and IL-6, decrease the incidence of PONV and receive additional analgesic and antiemetic effects, and achieve a better ROM and patient satisfaction, without increasing the risk of wound-related complications when compared with patients in Group A and B.

TKA is one of the most effective methods to treat knee diseases [1, 2]. However, surgical trauma following TKA often results in severe postoperative inflammation, which is associated with postoperative pain and PONV [8, 9]. As a result, the fast-track treatment of the patients was hampered postoperatively. Glucocorticoids are known for their analgesic, anti-inflammatory, and anti-emetic effects, although the mechanisms are unclear. In using corticosteroids, therefore, its pharmaceutical ingredients enter the surrounding tissue and reduce the inflammatory response at the site of surgical trauma, thus providing effective pain relief. Dexamethasone is a kind of synthetic glucocorticoid with high bioavailability and long acting time. In the current study, significant increases in CRP and IL-6 levels were found in the three treatment groups, however, CRP and IL-6 levels increased significantly less in Groups C and B compared with Group A at 24, 48, and 72 h. It was also observed to be lower in Group C than in Group B. Thus, our findings suggest that intravenous dexamethasone administered 1 h preoperatively and repeated at 6 h postoperatively reduced the postoperative inflammatory response compared to Groups A and B.

Compared with the level of pain preoperatively, pain declined after TKA among the three groups. As previously reported, the analgesic effect of glucocorticoid is achieved by inhibiting phospholipase, thus blocking the pathway of cyclooxygenase and lipoxygenase in the inflammatory chain reaction [29]. At the same time, it can also inhibit the level of bradykinin [30] in tissues and the release of neuropeptides from nerve endings [31], both of which may enhance the sense of injury in inflammatory tissues and surgical wounds. An Randomized Controlled Trial (RCT) performed by Koh et al. [22] prospectively evaluated 269 TKAs randomized to receive dexamethasone (10 mg) combined with ramosetron or ramosetron alone. These results suggest that patients with dexamethasone experienced lower pain and consumed fewer opioids during the 6 to 24 h postoperative. Similarly, another RCT study by Xu et al. [32] involving 108 TKA patients divided into 2 groups of 54 patients. The two treatment groups were either given two doses of 10 mg IV dexamethasone or placebo, the study found that the pain at rest and walking was lower at 24 h postoperatively in the dexamethasone group. The results in current study echo those findings. We showed a statistically significant reduction of the VAS score of pain in Group C than Group A at 24 and 48 h postoperatively and Group B at 24 h postoperatively. Furthermore, the requirements of oxycodone and pethidine hydrochloride were significantly lower in Group C.

The antiemetic effects of dexamethasone were well documented [17, 19, 22, 32] and supported in our study. However, the mechanism by which glucocorticoids relieve PONV is not fully understood. Its effect may be mediated by the inhibition of prostaglandin synthesis or the inhibition of endogenous opioid release [33]. The current study showed that two doses of dexamethasone were more effective in preventing the PONV and reducing the intensity of nausea than the single dose or without dexamethasone groups. The results are consistent with previous studies [10, 22, 32] suggesting that dexamethasone has a better antiemetic effect. In a recent meta-analysis of 3 randomized controlled trials [34], dexamethasone not only reduces postoperative pain level and opioid consumption within 48 h but also reduces postoperative PONV. Although few previous studies have investigated the antiemetic efficacy of two doses of low-dose dexamethasone after TKA. The current results as well as previous studies [10, 32] suggest that the use of multiple low doses of dexamethasone has a stronger antiemetic effect on the early stage of PONV than a single dose of dexamethasone. The results therefore suggest that the repeated administration of dexamethasone 6 h postoperatively can effectively reduce the incidence of PONV. Additionally, functional outcome assessments in our study demonstrated a better ROM in Group C. The outcomes might explain the significantly higher satisfaction ratings of patients in Group C at the time of discharge.

However, the optimal routes and doses of dexamethasone remain controversial. A range of doses from 4 to 10 mg in primary TKA has been established in most studies [22, 35]. Barnes et al. [36] suggested that glucocorticoids bind to intracellular glucocorticoid receptors, which are mainly mediated by the synthesis of transcriptional proteins. Therefore, the starting time of biological action is generally 1–2 h [29], and it was reasonable to give dexamethasone 1 h before the surgery so that it reached its plasma concentration at the beginning of resuscitation in this study. In addition, since early media-activated metabolic reaction occurs immediately after surgical incision [37], it is also reasonable to give dexamethasone 1 h before the surgery to obtain a potential therapeutic effect postoperatively. As previously reported [22, 27], patients still have to deal with pain and PONV with single-dose dexamethasone. Thus, we added an additional 10-mg dose of dexamethasone at 6 h postoperatively, assuming that it would have a meaningful clinical benefit.

Although TKA is associated with thrombosis [3, 38], and glucocorticoids are associated with wound healing and postoperative infection, previous studies using low or high doses of glucocorticoids have not shown that glucocorticoids are associated with these complications during the perioperative period [22, 32, 34]. In our study, we also found that the additional low dose of dexamethasone was given at 6 postoperatively, and no patients had an obvious adverse effect and/or complication during postoperative follow-up. It is known that synthetic glucocorticoids,such as dexamethasone, prescribed in daily care can induce hypertension. However, the exact mechanism by which glucocorticoids may induce hypertension is unclear [39]. Some evidence supports the fact that glucocorticoids may actually have nothing to do with elevated blood pressure in the first few weeks. Gabriel et al. [40] reported that 15.3% of 124 patients with rheumatoid polymyalgia prescribed glucocorticoid treatment for hypertension, and 26.3% of 57 patients who received non-steroidal anti-inflammatory drugs had hypertension. Furthermore, Fardet et al. [41] also sugessted that the increase of blood pressure during the first months of exposure to synthetic glucocorticoids seems clinically nonsignificant. At the same time, there was also no evidence that perioperative dexamethasone administration is associated with increased postoperative glucose levels (> 200 mg/dl) or higher maximum glucose levels in total joint arthroplasty [42]. Therefore, we believe that there is evidence to support that two doses of low-dose preoperative treatment with dexamethasone are safe in TKA procedures.

This study had some limitations. The follow-up period for assessing functional outcome was 24 h, 48 h and 72 h postoperatively; therefore, this study does not show the long-term effects of dexamethasone. Second, although this study showed a lower incidence of wound-related complications, a larger sample size is required to adequately assess the significant differences in adverse events. Third, two doses of low-dose dexamethasone showed better clinical results, but the best method and dose of dexamethasone and whether a further use is needed to produce additional results remain unclear.

## Conclusions

The administration of 10 mg dexamethasone 1 h before the surgery, and repeated at 6 h postoperatively can significantly reduce the level of postoperative CRP and IL-6 and the incidence of PONV, relieve pain, achieve an additional analgesic effect, and improve the early ROM compared with the other two groups in TKA.

## Abbreviations

BMI: Body mass index
CRP: C-reactive protein
DVT: Deep vein thrombosis
IL-6: Interleukin 6
LOS: Length of stay
PBV: Patient’s blood volume
PE: Pulmonary embolism
PONV: Postoperative nausea and vomiting
ROM: Range of motion
TKA: Total knee arthroplasty
VAS: Visual analogue scale
